# Relationship between Female University Students' Knowledge on Menstruation and Their Menstrual Hygiene Practices: A Study in Tamale, Ghana

**DOI:** 10.1155/2016/1056235

**Published:** 2016-07-25

**Authors:** Evans Paul Kwame Ameade, Helene Akpene Garti

**Affiliations:** ^1^Department of Pharmacology, School of Medicine and Health Sciences, University for Development Studies, Tamale, Ghana; ^2^Department of Community Nutrition, School of Allied Health Sciences, University for Development Studies, Tamale, Ghana

## Abstract

Positive perception about menstruation and good menstrual hygiene practice safeguards the health of postpubescent females by reducing their vulnerability to reproductive and urinary tract infections. Using a questionnaire, a cross-sectional study involving 293 randomly selected female undergraduate students in northern Ghana assessed the relationship between knowledge on menstruation and the practice of safe menstrual hygiene. Data collected was analyzed using GraphPad 5.01. This study found that although majority of respondents (73.4%) were aware of menstruation before menarche, most of them experienced fear and panic when it occurred. Mothers were the first to be informed when menstruation occurred, although teachers first provided them knowledge on menstruation. Respondents' knowledge on menstruation was average (57.3%) but their menstrual hygiene practice was good (80.2%). Age (*p* = 0.005) and course of study (*p* = 0.0008) significantly influenced respondents' knowledge on menstruation with older students as well as the medical and midwifery students being most knowledgeable. Muslim rather than Christian female students practiced better menstrual hygiene (*p* = 0.0001). Average knowledge score on menstruation indicated a deficit of knowledge on the anatomy and physiology of the female reproductive system. Increasing knowledge on menstruation had a positive and significant effect on practice of good menstrual hygiene.

## 1. Introduction

Menstruation, a unique event in the life of a developing girl child, is one of the milestones of puberty. It involves the cyclical shedding of the inner lining of the uterus which is controlled by the hormones produced by the hypothalamus and pituitary glands located in the brain [1]. The age at which women experience their first menstrual flow (menarche) varies widely across the world but generally most studies report that it occurs between ages of 13 and 15 years [2–7]. Although the age at which women stop menstruating is not the same in all nations, menopause is reported to usually occur between the ages of 45 and 50 years [2]. A woman therefore spends approximately 2100 days menstruating which is equivalent to almost 6 years of her reproductive life [8].

Whereas in some societies onset of menstruation is celebrated, it is the beginning of imposition of dietary and social restrictions at some other places [1, 9–11]. These sociocultural impositions during the period of menstruation make some menstruating females perceive this phenomenon not only as burdensome but also as an event that ushers in fear, disgust, and shame.

Provision of adequate knowledge on menstruation before menarche could make young females view menstruation as an important milestone in their lives and just a natural phenomenon. Parents and close relations are expected to be the foremost source of information on menstruation to young females but unfortunately in Africa, parent-child communication about sexually related matters is poor; hence most adolescents acquire sometimes incorrect information on the reproductive system from their friends [12].

Low knowledge on menstruation increases the risk of contracting reproductive tract infections as well as pelvic inflammatory diseases and urinary tract diseases among millions of women across the world, because they are unable to manage their menstrual periods well enough [1, 13]. Good menstrual hygiene management involves women or adolescent females using clean blood-absorbing materials which can be changed often in a secure place in privacy after which soap and water are available to wash hand and body as well as having access to secured used sanitary material disposal facility [14].

Several studies conducted among adolescent and pretertiary students across the world have rather shown inadequate knowledge of menstruation and poor menstrual hygiene practices [11, 15, 16]. Society considers persons with tertiary education to be knowledgeable in diverse fields and for students in health sciences, their knowledge related to reproductive systems such as menstruation would be highly considered. This study therefore assessed the knowledge of female undergraduate students in Schools of Medicine and Allied Health Sciences in the University for Development Studies in Tamale, Ghana, on menstruation and their practice of good menstrual hygiene. The study also assessed factors that could influence the knowledge of these students on menstruation as well as their practice of good menstrual hygiene.

## 2. Method

### 2.1. Study Design and Setting

This cross-sectional study conducted between March 2015 and April 2015 was among female undergraduate students studying Medicine, Nursing, Midwifery, Health Science Education, and Community Nutrition. The study setting was the Schools of Medicine and Health Sciences and Allied Health Sciences both of the University for Development Studies, Tamale. Tamale, the capital of the Northern region of Ghana and the fourth largest city in Ghana at 2010, had a population of 371,351 [17]. The instrument for this study was a semistructured questionnaire. This questionnaire was initially piloted among 20 students which ensured correction of ambiguous and inconsistent questions before it was administered for the actual data collection.

### 2.2. Study Variable Measurements

The knowledge of the students on menstruation was based on 10 questions relating to menstrual physiology, female anatomy, and menstrual hygiene. Menstrual hygiene practice was assessed using 6 questions. Each most appropriate or correct response scored one point whereas a wrong or do not know answer attracted no score. The overall maximum scores for knowledge and menstrual practice were 10 and 6 points, respectively. Overall knowledge scores converted to nearest whole numbers from 0 to 4 were adjudged as poor, 5 to 6 as average, and 7 to 8 as good, while those above 9 were adjudged as excellent. For each knowledge and menstrual hygiene practice question, correct mean score of 0.0 to below 0.5 (<50%) was poor, 0.5 to less than 0.7 (50.0% to <70.0%) was considered as average, above 0.7 to less than 0.9 (70.0% to <90.0%) was considered as good, and above 0.9 or 90% was excellent. For menstrual hygiene practice, overall score between 0 and 3 was considered poor while score above 3 was adjudged as good. The mean scores were recorded with their standard deviations (mean value ± standard deviation).

### 2.3. Ethical Consideration

Prior approval for this study and the procedures were obtained from the Ethics Committee of the School of Medicine and Health Sciences of the University for Development Studies. Verbal consent was obtained based on provision of adequate participant information that enhanced respondents' confidentiality in the research which increased their participation. Respondents were adequately informed that accepting to participate and completing the questionnaire indicated consent with an option of withdrawing from the research at any point.

### 2.4. Study Size Determination and Sampling Procedure

Study sample size was estimated using Cochran's correction formula for categorical data: *n*_1_ = *n*_0_/(1 + *n*_0_/population), where *n*_1_ is the required return sample size without estimated response rate factor and *n*_0_ is the required sample size which was calculated based on the assumption that 50% of respondents possess good knowledge of menstruation and also practiced good menstrual hygiene, sampling error is 5%, confidence interval is 95%, and the significant level *t*-value at alpha level of 0.05 is 1.96. The calculated return sample size without estimated response factor was therefore 384. With the study population of 990 and an estimated response rate of 70%, the drawn sample size of 389 was obtained for this study. The respondents were drawn using the sampling with replacement method. The number of respondents from a class in a study programme was obtained using a proportional approach based on the number of female students in each class. In each class, respondents were randomly chosen by picking from envelope pieces of paper with name and identity number of each female member of the class printed on it. The response rate for this study was 75.3% (*n* = 293/389).

### 2.5. Statistical Analysis

Data was entered into Microsoft Excel and analyzed using GraphPad Prism, Version 5.01 (GraphPad Software Inc., San Diego, CA). Associations between respondents' sociodemographic characteristics and their knowledge of menstruation and practice of good menstrual hygiene scores were assessed using the one-way ANOVA test as well as independent *t*-test, whichever is applicable. Test of association between age, knowledge of respondents about menstruation, and their practice of menstrual hygiene was determined using Pearson correlation coefficient. Statistical significance was assumed at *p* < 0.05 and at a confidence interval of 95%.

## 3. Results

### 3.1. Sociodemographic Profile

The sociodemographic profile of the respondents is as shown in Table 1. In this study, the majority, 221 (75.4%), were between ages of 20 and 25 years, experienced menarche between ages of 13 and 15 (mean age of menarche = 13.66 ± 1.87 years), were Christians, 208 (71.0%), and spent their vacation in urban areas of Ghana, 181 (61.8%). Most of the students were studying Nursing, 99 (33.8%), and were in the second year, 111 (37.9%). At menarche, most respondents, 126 (43.0%), stayed in a self-contained accommodation, indicative of their parents and guardians belonging to the middle social class. Majority, 181 (61.8%), of the students currently live in urban areas of Ghana.

### 3.2. Level of Awareness of Respondents of Menstruation at Menarche and Their Reaction to First Menstrual Blood

Majority, 215 (73.4%), of the respondents were aware of menstruation before the onset of menarche with the teachers being the earliest source of information for majority of them, 159 (54.3%). Mothers were however the first persons the majority, 181 (61.8%), discussed menarche with rather than the teachers who were the least consulted, 5 (1.7%). Majority, 198 (72.5%), expressed satisfaction with the level of education on menstruation they got from persons they first discussed their first menstrual episode with. Most, 105 (38.5%), of the respondents were struck by fear and panic on seeing blood flowing from their genitals for the first time at menarche.  Table 2 shows respondents' level of awareness of menstruation at menarche and subsequent reaction to menarche.

### 3.3. Menstrual Hygiene Practices of Respondents

During the first year after menarche, a fifth of the respondents, 61 (20.8%), used other sanitary materials to absorb their menstrual blood rather than commercially available disposable sanitary pads. However, currently all respondents use sanitary pad for management of their menstrual flow. Majority, 169 (57.7%), change their sanitary pads twice a day and 203 (69.3%) clean their genitals after urinating during menstruation with most, 95 (46.8%), of them using only toilet tissue for the cleaning. The used sanitary pads were disposed into refuse bins by majority, 173 (59.0%), of the respondents. Whereas only 14 (4.8%) skipped bathing with soap and water on the first day of menses, majority, 192 (65.5%), did not increase the number of baths taken during their period of menstruation. Mean menstrual hygiene practice scores of more than 0.7 or 70% were recorded in relation to use of sanitary pad (1.0), changing sanitary pad at least twice or more a day during the menstrual period (0.89 ± 0.31), disposal of used sanitary pad at appropriate sites (0.93 ± 0.25), and not skipping bathing with soap and water on first day of menses (0.95 ± 0.22). The worst menstrual hygiene practice by the respondents was in relation to not increasing the number of baths during menstruation (0.35 ± 0.48). The overall menstrual practice score of the respondents was 4.81 ± 0.90/6 or 80.2%.  Table 3 shows the menstrual hygiene practices of respondents.

### 3.4. Knowledge of Respondents on Menstruation

Table 4 shows respondents' overall mean knowledge score in this study to be 5.73 ± 1.56 or 57.3%. Questions for which mean knowledge scores were more than 0.7 or 70% were about the definition of menstruation, 0.93 ± 0.26 (92.8%); normal interval between menstrual cycles, 0.76 ± 0.43 (75.8%); whether there is a period in a menstrual cycle when a woman is most fertile, 0.79 ± 0.41 (78.5%); and whether poor menstrual hygiene can lead to infections, 0.96 ± 0.20 (95.9%). The respondents scored least on the normal age at menarche, 0.22 ± 0.41 (21.5%), what was responsible for menstruation, 0.26 ± 0.44 (26.3%), normal number of days of menstrual flow, 0.40 ± 0.49 (39.6%), and whether a woman can ever be pregnant during menses, 0.29 ± 0.46 (29.4%). The score for the knowledge on the source of the menstrual blood being the uterus was 0.56 ± 0.50 (55.9%) but up to 44.0% did not know or stated other organs such as vagina, ovaries, unfertilized ovum, and fallopian tubes as the origin of the menstrual blood.

### 3.5. Relationship between Sociodemographic Characteristics and Respondents' Score of Knowledge on Menstruation and Menstrual Hygiene Practice

Table 5 shows the effect of sociodemographic characteristics of the respondents on their knowledge on menstruation and practice of menstrual hygiene. In this study, females older than 25 years were significantly more knowledgeable about menstruation than their younger colleagues (6.49 versus 5.60–5.73; *p* = 0.005) and also practiced better menstrual hygiene but the difference was not significant. Christians scored higher mean knowledge score than followers of Islam (5.83 versus 5.51) but their mean menstrual hygiene practice score was significantly lower than the Muslims (4.70 versus 5.15; *p* = 0.0001). ANOVA analysis showed a significant difference in knowledge scores among the students based on their courses of study (*p* = 0.0008) with the medical (6.11) and Midwifery (6.19) students scoring more than 6 out of 10 while Community Nutrition students obtained the least mean knowledge score of 5.08. Course of study did not however influence students' menstrual hygiene practice. As students move to higher levels in their study, their knowledge on menstruation and their menstrual hygiene practice increased since it was the fourth-year students who obtained the highest knowledge (5.93 versus 5.57–5.84) and menstrual hygiene practice (5.07 versus 4.64–4.96) scores. There was however a significant difference in menstrual hygiene practice based on year of study (*p* = 0.028). Socioeconomic situation of respondents at menarche (indicated by the type of accommodation occupied at menarche) and the living area of respondents did not influence their knowledge on menstruation and their practice of menstrual hygiene.

Table 6 shows the association between continuous variables of age, respondents' knowledge on menstruation, and their menstrual hygiene practices. Whereas there was a weak positive but significant relationship between the age of respondents and their knowledge of menstruation (*r* = 0.14; *p* = 0.0195), it was not so with their menstrual hygiene practices. There was also a positive and significant association between the knowledge of respondents about menstruation and their practice of good menstrual hygiene (*r* = 0.26; *p* < 0.0001).

## 4. Discussion

Hormonal changes during puberty set into motion the transformation of a girl child to a woman who had attained sexual maturity which is accompanied by psychological, cognitive, and physical changes [18]. Menstruation, which is one of the milestones of puberty in a girl, involves the cyclical shedding of the inner lining of the uterus which is controlled by the hormones of the hypothalamopituitary axis [1]. Better appreciation of and attitude towards menstruation are achieved when the girl child is aware or knowledgeable about menstruation [19]. According to Auemaneekul et al. [20] and Bhore and Kumbhar [21], females with greater knowledge on menstruation were better in practicing good menstrual hygiene. Several studies reported low to average levels of awareness of menstruation at menarche between 36.9% and 67.5% [1, 13, 16, 22–24]. This study however recorded 73.4% of respondents stating that they knew what menstruation was before menarche. Teachers were stated by majority (54.3%) of respondents to be their foremost source of information about menstruation not mothers as reported in several previous studies [1, 13, 15, 16, 23, 24]. One possible reason for teachers rather than mothers being the first source of information on menstruation in this study is because, in Ghana, pupils in primary five are taught human organs and their functions in Natural Science lessons and the teachers would have introduced menstruation when teaching organs of the reproductive system. Furthermore, in the African societal setup, parents shy away from discussing issues related to sex and reproduction with their daughters [9]. This study however found that when menstruation finally occurred, majority of respondents first discussed it with their mothers (61.8%). Mothers were first notified of menarche because they would have to provide the menstruating girl with sanitary materials to handle the flow and most importantly females consider menstruation-related matters as challenges best discussed with other females preferably mothers, rather than males [25, 26]. Lack of trust in teachers was reported by female students in Sierra Leone to be one of the reasons they were reluctant to discuss their menstruation issues with their teachers [26]. The situation where males form the majority of teacher population in educational institutions in Sub-Saharan Africa as well as South and West Asia makes teachers even more unattractive to these female students [27]. In Ghana, the female teacher percentages in primary and secondary schools were 33.0% and 22.0%, respectively, as at 2007 when most respondents in this study were at those levels of education [27]. With this low number of female teachers, these female students would rather inform their mothers or elder female relatives about menarche rather than their male teachers.

Although almost three-fourths of respondents might have heard of menstruation before menarche, 38.5% exhibited fear and panic when they first saw blood flowing from their genitals at menarche. Negative reactions of fear, discomfort, anxiety, and shame as reported in studies in other parts of the world could be attributed to the low knowledge on menstruation among the young adults [8, 23, 28]. Several studies among secondary school students showed that knowledge about menstruation was mostly average or inadequate [11, 21–23].

Undergraduate students pursuing health related programmes such as respondents in this study were expected to possess good knowledge on menstruation so the mean knowledge score of 57.3% recorded in this study can best be described as average. Whereas majority of respondents in this study correctly defined menstruation and agreed that poor menstrual hygiene may cause infections, less than a third (26.3%) knew that hormones are responsible for the menstrual cycle, a score worse than the 55.0% recorded among secondary school students in Nepal [15]. Furthermore, three-fourths of respondents did not know or stated the normal age of menarche to be 12 years or below. The normal ages of menarche stated by about three-fourths (72.5%) of these females were lower than their own recall age at menarche. Meanwhile, the recall age at menarche from this study was 13.66 years lower than 13.98 years found by Adadevoh et al. [29], which was Ghana's first survey on age at menarche. The age at menarche and the cause of menstruation are basic important information that girls should know so they can better appreciate menstruation and the accompanying physiological and psychological changes in the life of a woman. The discord between the perceived age at menarche and the average normal age at menarche and for more than half (51.9%) to state that normal menstrual flow should last more than 5 days can be a source of anxiety for many of these female students. In this study, two factors, namely, the age of respondents (*p* = 0.005) and the course of study (*p* = 0.0008), significantly influenced a respondent's knowledge on menstruation. The Midwifery class which scored the highest knowledge score had some older but lower certificated students who have enrolled in the university to further their studies. The working environments of these practicing midwives may possibly have provided them with extra knowledge on menstruation which might have been the reason for their relatively higher knowledge scores.

Poor menstrual hygiene increases the vulnerability of postpubescent females to reproductive tract, urinary tract, and perineum infections [30, 31]. This study found a positive and significant association between the knowledge of respondents on menstruation and their practice of good menstrual hygiene management (*r* = 0.25; *p* < 0.0001) which is contrary to a study in India by Shanbhag et al. [23].

Despite the average knowledge on menstruation exhibited by the undergraduate female students in this study, their practice of menstrual hygiene score of 80.2% was good. Earlier studies especially in developing countries reported poor menstrual hygiene practices [8, 22, 32]. In this study, all the females used disposable sanitary pads but other studies have some females still using other materials such as reuseable pieces of cloth for absorbing menstrual blood [8, 26]. All respondents use disposable sanitary pads because majority come from urban areas and have parents of the middle class; hence accessibility and affordability of sanitary pads should not be a challenge. Respondents who change their sanitary pad twice in the day were in the majority in this study similar to that reported in other countries such as Nepal [15] and India [8]. Cleaning of the genitals regularly after urinating prevents unpleasant odour as excess blood would not stay long enough in the skin between the labia or crust around the opening of the vagina to create the odour. The regular cleaning of genitals during menstruation recorded a low practice rate of 24.8% in Pokhrel et al. [8] study but respondents in this study scored 69.3%. Most of those who clean their genitals after urinating use toilet tissue only because taps in the washrooms on campus do not always flow and also the washrooms were not designed to make washing convenient. Those who do not clean the genitals did not find it necessary or for heavy bleeding females the fear of spilling blood prevents them from attempting any cleaning while on campus. The need for more regular baths during menses enables the female to clean the genitals with water more often but majority of females in this study did not find it necessary to increase the number of daily baths which was their worst menstrual hygiene practice score (34.5%). This study, just as that by Shanbhag et al. [23], did not report residential status and living area influencing the practice of safe menstrual hygiene. Among the sociodemographic characteristics of respondents in this study, religion and year of study are the factors that were associated with the female's practice of safe menstrual hygiene. Anusree et al. [11] reported effect of religion on menstrual hygiene practice with Muslims doing better than followers of other religions. This study also found Muslims scoring significantly better than Christians in safe menstrual hygiene practice. According to Dunnavant and Roberts [33] women whose religions prescribed various rituals and prohibited them from some activities have more negative attitude towards menstruation. Islam is one of such religions which consider menstruation a period of impurity and prohibition from several religions activities [33, 34] which could be the reason female Muslims would put in more effort to attain better menstrual hygiene than their Christian colleagues.

Several limitations associated with this study are worth stating. This study involved the use of self-administered questionnaires rather than interviews; hence reliability of answers could not be verified. Again, the menstrual hygiene practice score may contain some social desirability biases since some respondents may state they practice safe menstrual hygiene but do otherwise. This study was undertaken among female students of one of the campuses of the University for Development Studies. The results of this study can therefore not be generalized for all females in Ghana. The use of simple random sampling removed bias in relation to the selection of respondents which invariably is one of the strengths in this study.

## 5. Conclusion

Female university students possessed average knowledge on menstruation but they practiced good menstrual hygiene. There was a positive and significant association between knowledge of students on menstruation and their practice of good menstrual hygiene. Of all the sociodemographic factors, age and course of study of students positively influenced their knowledge about menstruation, while religion and year of study were associated with their practice of safe menstrual hygiene. The mean age at menarche was 13.66 years which means that education on menstruation and menstrual hygiene management should start from upper primary so that females would be well informed on this normal phenomenon before menarche.

## Figures and Tables

**Table 1:** Sociodemographic characteristics of the respondents.

Variable	Subgroups	Number of respondents	Percentages
Age (years)	<20	33	11.3
Mean age = 23 ± 5.07	20–25	221	75.4
Median = 22 years	>25	39	13.3

Age of menarche	<13	83	28.3
Mean age = 13.7 ± 1.87	13–15	161	54.0
Median = 14 years	>15	49	16.7

Course of study	Community Nutrition	52	17.7
	Health Science Education	29	9.9
	Medicine	54	18.4
	Midwifery	59	20.1
	Nursing	99	33.8

Year of study	1	67	22.9
	2	111	37.9
	3	70	23.9
	4	45	15.3

Religious affiliation^*∗*^	Christianity	208	71.0
	Islam	79	27.0

Type of accommodation at menarche^*∗*^	Single room	46	15.7
	Chamber and hall	55	18.8
	Several rooms in a compound house	52	17.7
	Self-contained apartment	126	43.0
	Mansion	10	3.4

Area of residence during vacation^*∗*^	Urban area	181	61.8
	Suburban	88	30.0
	Rural	21	7.2

^*∗*^There are missing values so percentages do not add up to 100 but are considered valid.

**Table 2:** Respondents' level of awareness of menstruation at menarche and their reactions on seeing their first menstrual blood.

Variable	Subgroup	Number of respondents	Percentage
Did you know of menstruation before it started?	Yes	215	73.4
	No	71	24.2

First source of information on menstruation	Teachers/school	159	54.3
	Elder sister	7	2.4
	Mother	63	21.5
	Friends	19	6.5
	Others	10	3.4

Reaction on seeing blood flow from your genitals the first time (*n* = 273)	Discomfort	8	2.9
	Surprise	13	4.8
	Shyness	5	1.8
	Fear and panic	105	38.5
	Sadness	10	3.7
	Indifference	92	33.7
	Happiness	27	9.9
	Confusion	13	4.8

Who was the first person you discussed your menstruation with?	Teacher	5	1.7
	Elder sister	34	11.6
	Mother	181	61.8
	Father	9	3.1
	Friends	15	5.1
	Other relatives	16	5.5
	Nobody	20	6.8

Did the person you first discussed your menarche with educate you enough on menstruation (*n* = 276)?	Yes	198	72.5
	No	65	23.8

**Table 3:** Menstrual hygiene practices (MHP) scores of respondents.

Variable	Subgroups	Number of respondents (%)	MHP score (mean ± SD)	MHP score (%)
Sanitary material used in first year of menstruation	Sanitary pad	229 (78.2%)	NA	NA
	Toilet tissue	30 (10.2%)		
	Reuseable cloth	27 (9.2%)		
	Others	4 (1.4%)		

Current sanitary material	Sanitary pad	293 (100.0%)	1.0 ± 0.0	100.0

How often sanitary pads are changed^*∗*^ Mean = 2.354 ± 0.686 Median = 2	Once	18 (6.1%)	0.89 ± 0.31	89.4
	Twice	169 (57.7%)		
	Thrice	87 (29.7%)		
	Four or more	17 (5.8%)		

Do you clean your genitals after urinating during menstruation?	Yes	203 (69.3%)	0.69 ± 0.46	69.3
	No	87 (29.7%)		

Materials for cleaning genitals after urinating	Water and tissue	18 (8.9%)	NA	NA
	Water only	78 (38.4%)		
	Tissue only	95 (46.8%)		
	Others	6 (3.0%)		

Method or place of disposal of waste sanitary pad	Toilet pit	15 (5.1%)	0.93 ± 0.25	93.2
	Refuse dump	24 (8.2%)		
	Refuse bin	173 (59.0%)		
	Bury	7 (2.4%)		
	Burn	54 (18.4%)		

Do you skip bathing with soap and water on first day of menses (*n* = 292)	Yes	14 (4.8%)	0.95 ± 0.22	94.5
	No	278 (94.9%)		

Number of times of bathing increases during menstruation	Yes	101 (34.5%)	0.35 ± 0.48	34.5
	No	192 (65.5%)		

Menstrual practice score (maximum = 6)			4.81 ± 0.90	80.2

SD: standard deviation. ^*∗*^Change of sanitary pad twice a day was considered adequate for persons who had light or moderate menstrual flow. Any respondent who had heavy menses should have changed sanitary pad at least thrice a day for her menstrual hygiene practice to be considered good for a score of 1 point to be awarded.

**Table 4:** Knowledge of respondents on menstruation (*n* = 293).

Variable	Subgroup	Number of respondents	Correct knowledge score	Percentage of correct scores
Definition of menstruation	Correct^#^	272 (92.8%)	0.93 ± 0.26	92.8
	Incorrect	21 (7.2%)		

What is the normal age(s) of menarche (years)^*∗*^	12 or less	175 (59.7%)		21.5
	13–15^c^	63 (21.5%)	0.22 ± 0.41	
	>15	2 (0.7%)		
	Do not know	53 (18.1%)		

What is responsible for menstruation?	Hormones^c^	77 (26.3%)	0.26 ± 0.44	26.3
	Incorrect	154 (52.6%)		
	Do not know	62 (21.2%)		

Source of menstrual blood	Inner lining of uterus^c^	164 (55.9%)	0.56 ± 0.50	55.9
	Incorrect	60 (20.5%)		
	Do not know	69 (23.5%)		

Normal number of days for menstrual flow	2 or less	1 (0.3%)		39.6
	3–5^c^	116 (39.6%)	0.40 ± 0.49	
	>5	152 (51.9%)		
	Do not know	24 (8.2%)		

Normal interval between menstrual cycles^*∗*^	Correct (21–35 days)^c^	222 (75.8%)	0.76 ± 0.43	75.8
	Incorrect	26 (8.9%)		
	Do not know	45 (15.4%)		

Is there a period women are most fertile?	Yes^c^	230 (78.5%)	0.79 ± 0.41	78.5
	No	36 (12.3%)		
	Do not know	27 (9.2%)		

Can woman ever be pregnant during menstrual flow?	Yes^c^	86 (29.4%)	0.29 ± 0.46	29.4%
	No	144 (49.1%)		
	Do not know	63 (21.5%)		

Normal menopausal age^*∗*^	Correct (45–50 years)^c^	169 (57.7%)	0.58 ± 0.50	57.7
	Incorrect	97 (33.1%)		
	Do not know	27 (9.2%)		

Poor menstrual hygiene can lead to infections	Yes^c^	281 (95.9%)	0.96 ± 0.20	95.9
	No	6 (2.0%)		
	Do not know	6 (2.0%)		

Mean knowledge score (maximum = 10)			5.73 ± 1.56	57.3

^*∗*^If a respondent gave a range of values for an answer, the lower value was chosen for knowledge score calculations. ^c^Most appropriate answer. ^#^Definition should be “monthly flow of blood through the vagina” or statements similar to this.

**Table 5:** Relationship between respondents' sociodemographic characteristics and their knowledge on menstruation and menstrual hygiene practice scores.

Variable	Subgroups	Knowledge score (mean ± SD)	*p* value	MHP score (mean ± SD)	*p* value
Age (years)	<20	5.73 ± 1.55	0.005^*∗*^	4.73 ± 1.07	0.83
	20–25	5.60 ± 1.56		4.82 ± 0.88	
	>25	6.49 ± 1.41		4.85 ± 0.88	

Age of menarche	<13	5.70 ± 1.52	0.96	4.78 ± 0.93	0.15
	13–15	5.76 ± 1.60		4.76 ± 0.92	
	>15	5.71 ± 1.50		5.04 ± 0.74	

Religious affiliation	Christianity	5.83 ± 1.57	0.12	4.70 ± 0.91	0.0001^*∗*^
	Islam	5.51 ± 1.54		5.15 ± 0.79	

Course of study	Community Nutrition	5.08 ± 1.82	0.0008^*∗*^	4.73 ± 0.89	0.56
	Health Science Education	5.83 ± 1.23		4.83 ± 0.93	
	Medicine	6.11 ± 1.48		4.93 ± 0.89	
	Midwifery	6.19 ± 1.69		4.68 ± 0.96	
	Nursing	5.58 ± 1.33		4.87 ± 0.87	

Year of study	One	5.57 ± 1.59	0.59	4.64 ± 1.01	0.028^*∗*^
	Two	5.69 ± 1.50		4.72 ± 0.92	
	Three	5.84 ± 1.59		4.96 ± 0.88	
	Four	5.93 ± 1.64		5.07 ± 0.58	

Type of accommodation at menarche	Single room	5.72 ± 1.68	0.67	4.48 ± 0.94	0.067
	Chamber and hall	5.55 ± 1.37		4.96 ± 0.86	
	Several rooms in a compound house	6.00 ± 1.74		4.89 ± 0.90	
	Self-contained apartment	5.75 ± 1.44		4.83 ± 0.88	
	Mansion	5.60 ± 2.55		5.00 ± 0.94	

Area of residence during vacation	Urban area	5.73 ± 1.52	0.52	4.83 ± 0.85	0.85
	Suburban	5.85 ± 1.73		4.77 ± 1.00	
	Rural	5.43 ± 1.12		4.86 ± 0.85	

^*∗*^Statistically significant.

**Table 6:** Correlation between continuous variables of age, respondents' scores on knowledge on menstruation, and their menstrual hygiene practices.

Variable 1	Variable 2	Pearson correlation coefficient (*r*)	*p* value
Knowledge on menstruation	Menstrual hygiene practices	0.26	<0.0001^*∗*^
Age of respondents	Knowledge on menstruation	0.14	0.0195^*∗*^
Age of respondents	Menstrual hygiene practices	−0.003	0.95

^*∗*^Statistically significant.
